# {2-[(9,9-Dihexyl­fluoren-2-yl)carbon­yl]phen­yl}(4-meth­oxy­phen­yl)methanone

**DOI:** 10.1107/S1600536812026773

**Published:** 2012-06-20

**Authors:** P. Narayanan, K. Sethusankar, Meganathan Nandakumar, Arasambattu K. Mohanakrishnan

**Affiliations:** a Department of Physics, RKM Vivekananda College (Autonomous), Chennai 600 004, India; b Department of Organic Chemistry, University of Madras, Guindy Campus, Chennai 600 025, India

## Abstract

In the title compound, C_40_H_44_O_3_, the fluorene ring system is essentially planar, with a maximum deviation of 0.075 (3) Å, and forms dihedral angles of 70.62 (8) and 70.31 (8)° with the mean planes of the central benzene ring and the meth­oxy­phenyl ring, respectively. Both the hexyl side chains have different conformations, *i.e*. an *anti*–*gauche*–*anti*–*gauche* conformation with C—C—C—C torsion angles of −169.3 (2), 74.2 (4), −178.0 (3) and −76.0 (6)° for one hexyl side chain and an *anti*–*anti*–*anti*–*gauche* conformation with C—C—C—C torsion angles of −177.9 (2), −176.5 (3), 171.7 (4) and 80.4 (9)° for the other. Four C atoms in one and two C atoms in the other hexyl side chains are each disordered over two sets of sites, with occupancy factors of 0.761 (3):0.239 (3) and 0.660 (6):0.340 (6). In the crystal, mol­ecules are *via* pairs of C—H⋯O hydrogen bonds, forming inversion dimers and resulting in *R*_2_^2^(28) graph-set motifs.

## Related literature

For the uses and biological importance of diketones, see: Saragi *et al.* (2004 ▶); Beulter *et al.* (2007 ▶). For related structures, see: Narayanan *et al.* (2011 ▶); Schollmeyer & Detert (2011 ▶). For distorted conformations, see: Judas *et al.* (1995 ▶). For graph-set notation, see: Bernstein *et al.* (1995 ▶). 
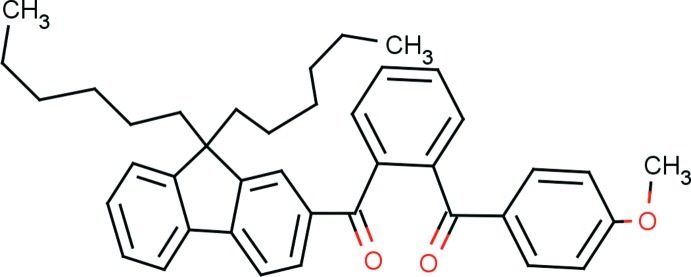


## Experimental

### 

#### Crystal data

C_40_H_44_O_3_
*M* *_r_* = 572.75
Monoclinic, 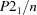

*a* = 16.7593 (10) Å
*b* = 11.4989 (6) Å
*c* = 17.146 (1) Å
β = 90.449 (2)°
*V* = 3304.2 (3) Å^3^
*Z* = 4
Mo *K*α radiation
μ = 0.07 mm^−1^
*T* = 293 K
0.30 × 0.25 × 0.20 mm

#### Data collection

Bruker Kappa APEXII CCD diffractometer
Absorption correction: multi-scan (*SADABS*; Bruker, 2008 ▶) *T*_min_ = 0.979, *T*_max_ = 0.986
28941 measured reflections
5850 independent reflections
3875 reflections with *I* > 2σ(*I*)
*R*_int_ = 0.037

#### Refinement

*R*[*F* ^2^ > 2σ(*F* ^2^)] = 0.050
*wR*(*F* ^2^) = 0.153
*S* = 1.02
5850 reflections
415 parameters
16 restraints
H-atom parameters constrained
Δρ_max_ = 0.23 e Å^−3^
Δρ_min_ = −0.32 e Å^−3^

### 

Data collection: *APEX2* (Bruker, 2008 ▶); cell refinement: *SAINT* (Bruker, 2008 ▶); data reduction: *SAINT*; program(s) used to solve structure: *SHELXS97* (Sheldrick, 2008 ▶); program(s) used to refine structure: *SHELXL97* (Sheldrick, 2008 ▶); molecular graphics: *ORTEP-3* (Farrugia, 1997 ▶); software used to prepare material for publication: *SHELXL97* and *PLATON* (Spek, 2009 ▶).

## Supplementary Material

Crystal structure: contains datablock(s) global, I. DOI: 10.1107/S1600536812026773/pv2555sup1.cif

Structure factors: contains datablock(s) I. DOI: 10.1107/S1600536812026773/pv2555Isup2.hkl

Supplementary material file. DOI: 10.1107/S1600536812026773/pv2555Isup3.cml

Additional supplementary materials:  crystallographic information; 3D view; checkCIF report

## Figures and Tables

**Table 1:** Hydrogen-bond geometry (Å, °)

*D*—H⋯*A*	*D*—H	H⋯*A*	*D*⋯*A*	*D*—H⋯*A*
C26—H26⋯O2^i^	0.93	2.58	3.470 (3)	160

Symmetry code: (i) 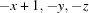.

**Fig. 1.**
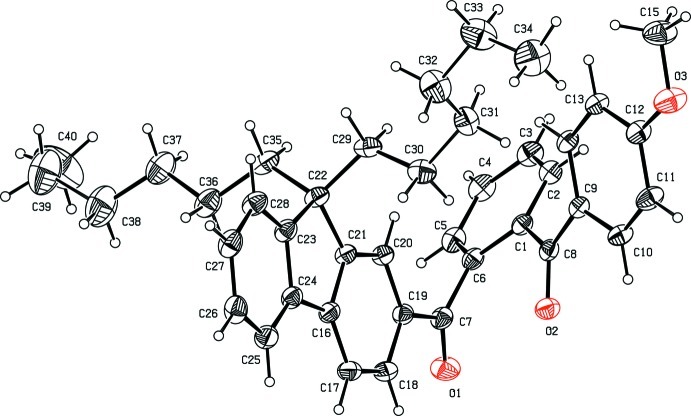
The molecular structure of the title compound showing only the atoms representing major fractions of the disordered dihexyl side chains, with the atom numbering scheme. The displacement ellipsoids are drawn at 30% probability level.

**Fig. 2.**
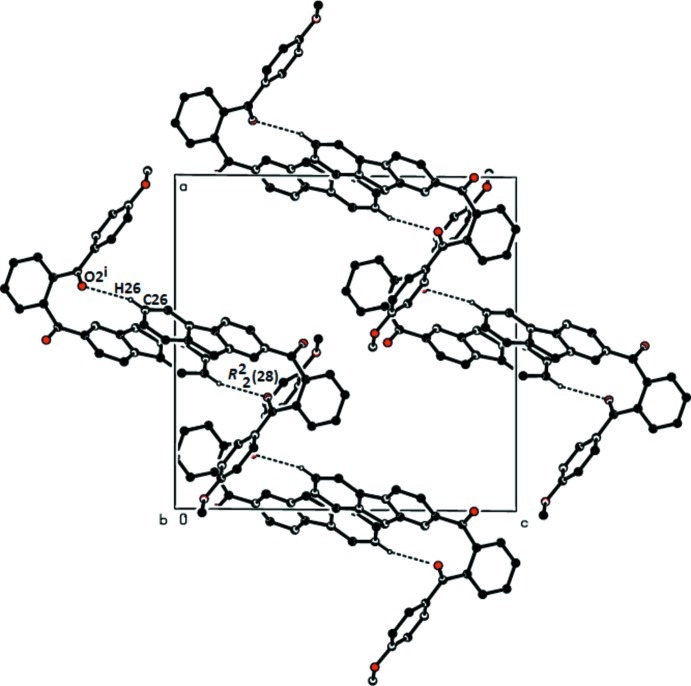
The crystal packing of the title compound, viewed down the a-axis, showing the centrosymmetric dimer via C—H···O hydrogen bonds. The dihexyl side chains and H atoms not involved in hydrogen bonding have been excluded for clarity.

## supplementary crystallographic information

### Comment

The fluorene derivatives have attracted much attention due to their potential utilities in organic light emitting devices, organic photo transistors, nonlinear optics and photochromic materials (Saragi *et al.*, 2004). They also possess antimalerial activity (Beulter *et al.*, 2007). In view of these important properties, the crystal structure of the title compound has been determined and reported in this article.

The title compound (Fig. 1), comprises a benzene ring attached to a diketone, a methoxy phenyl ring and a dihexyl fluorene ring system. The carbonyl oxygen atoms are significantly deviated [O1 = -1.0104 (17) Å and O2 = -0.8195 (14) Å] from the central benzene ring (C1–C6). The molecule possess a distorted *S*–conformation, with the participation of the atoms (C9/C8/C1/C6/C7/C19/O1/O2), as evidenced by the dihedral angle of 57.85 (8) ° between the two acetone planes defined by (C9/C8/C1/O2) and (C6/C7/C9/O1) (Judas *et al.*, 1995).

The fluorene ring system is essentially planar with a maximum deviation of 0.075 (3) Å for C27 atom. It forms dihedral angles of 70.62 (8) and 70.31 (8) ° with the central benzene ring (C1–C6) and methoxy phenyl ring (C9–C14), respectively. In the fluorene ring system, the five membered cyclopentadiene ring forms the dihedral angles of 1.61 (11) ° and 2.84 (12) ° with fused benzene rings (C16–C21) and (C23–C28), respectively.

Both the hexyl side chains have different conformations. One of the hexyl side chains has *anti*–*gauche*–*anti* –*gauche* conformation with C–C–C–C torsion angles -169.3 (2), 74.2 (4), -178.0 (3) and -76.0 (6) °. The other hexyl side chain has the conformation *anti*–*anti*–*anti*–*gauche* with C–C–C–C torsion angles -177.9 (2), -176.5 (3), 171.7 (4) and 80.4 (9) °. The hexyl side chains are disordered over two sets of sites, with the occupancy factors of 0.761 (3)/0.239 (3) and 0.660 (6)/0.340 (6).

In the crystal packing, molecules are linked into centrosymmetric dimers *via* C26—H26···O2^i^ hydrogen bonds, resulting in *R*^2^_2_(28) graph-set motifs (Bernstein *et al.*, 1995) (Table 1 and Fig. 2). The bond lengths and bond angles in the title compound agree with the corresponding bond lengths and angles reported for closely related compounds (Narayanan *et al.*, 2011); Schollmeyer & Detert, 2011).

### Experimental

To a solution of benzo[*c*]furan (0.50 g, 0.898 mmol) in dichloromethane (15 ml), meta-chloroperoxybenzoic acid (*m*-CPBA) (0.23 g, 1.347 mmol) was added and the reaction mixture was stirred at room temperature for 5 minutes. It was then poured into saturated sodium bicarbonate solution, extracted with dichloromethane (3x30 ml). The combined organic extract was washed with water (2x30 ml) and dried (Na_2_SO_4_). Removal of solvent followed by column chromatographic purification (silica gel, 5% ethyl acetate/hexane) afforded the diketone as a pale yellow solid (yield = 0.46 g, 81%). The product was dissolved in chloroform and heated for two minutes. The resulting solution was subjected to crystallization by slow evaporation of the solvent resulting in single crystals suitable for XRD studies. M.P. = 403–404 K.

### Refinement

In the dihexyl side chain atoms (C31/C32/C33/C34) and (C39/C40) were disordered over two sets of sites with the occupancy factors of 0.761 (3)/0.239 (3) and 0.660 (6)/0.340 (6). The bondlengths of the both major and minor components are restrained to a standard value using the commands DFIX, EADP (Sheldrick, 2008) and s.u. of 0.01 Å. The H atoms were placed at calculated positions in the riding model approximation with C—H = 0.93, 0.96 and 0.97 Å for aryl, methyl and methylene H-atoms, respectively, with *U*_iso_(H) = 1.5*U*_eq_(methyl C) and 1.2*U*_eq_(non-methyl C). The rotation angles for methyl groups were optimized by least squares.

### Crystal data

**Table:**

C_40_H_44_O_3_	*F*(000) = 1232
*M**_r_* = 572.75	*D*_x_ = 1.151 Mg m^−^^3^
Monoclinic, *P*2_1_/*n*	Mo *K*α radiation, λ = 0.71073 Å
Hall symbol: -P 2yn	Cell parameters from 5850 reflections
*a* = 16.7593 (10) Å	θ = 2.1–25.1°
*b* = 11.4989 (6) Å	µ = 0.07 mm^−^^1^
*c* = 17.146 (1) Å	*T* = 293 K
β = 90.449 (2)°	Block, colourless
*V* = 3304.2 (3) Å^3^	0.30 × 0.25 × 0.20 mm
*Z* = 4	

### Data collection

**Table:**

Bruker Kappa APEXII CCD diffractometer	5850 independent reflections
Radiation source: fine-focus sealed tube	3875 reflections with *I* > 2σ(*I*)
Graphite monochromator	*R*_int_ = 0.037
ω and φ scans	θ_max_ = 25.1°, θ_min_ = 2.1°
Absorption correction: multi-scan (*SADABS*; Bruker, 2008)	*h* = −18→19
*T*_min_ = 0.979, *T*_max_ = 0.986	*k* = −13→13
28941 measured reflections	*l* = −20→20

### Refinement

**Table:**

Refinement on *F*^2^	Primary atom site location: structure-invariant direct methods
Least-squares matrix: full	Secondary atom site location: difference Fourier map
*R*[*F*^2^ > 2σ(*F*^2^)] = 0.050	Hydrogen site location: inferred from neighbouring sites
*wR*(*F*^2^) = 0.153	H-atom parameters constrained
*S* = 1.02	*w* = 1/[σ^2^(*F*_o_^2^) + (0.0686*P*)^2^ + 1.0846*P*] where *P* = (*F*_o_^2^ + 2*F*_c_^2^)/3
5850 reflections	(Δ/σ)_max_ < 0.001
415 parameters	Δρ_max_ = 0.23 e Å^−^^3^
16 restraints	Δρ_min_ = −0.32 e Å^−^^3^

### Special details

**Table:**

Experimental. ^1^H-NMR (300 MHz, CDCl_3_): δ 7.65 – 7.54 (m, 10H, ArH), 7.28 (broad s, 7H, ArH), 6.76 (d, J = 8.4 Hz, 2H, ArH), 3.75 (s, 3H, OCH_3_), 1.88 – 1.83 (m, 4H, CH_2_), 1.05 – 0.95 (m, 12H, CH_2_), 0.71 – 0.48 (m, 10H, CH_2_CH_3_). ^13^C-NMR (75 MHz, CDCl_3_): δ 196.7, 195.4, 163.6, 152.1, 150.8, 146.0, 140.0, 139.8, 135.8, 132.3, 130.3, 129.8, 129.7, 129.2, 128.4, 127.0, 124.0, 123.1, 120.7, 119.2, 113.6, 55.4, 55.2, 40.1, 31.5, 29.7, 23.7, 22.6, 14.0.
Geometry. All e.s.d.'s (except the e.s.d. in the dihedral angle between two l.s. planes) are estimated using the full covariance matrix. The cell e.s.d.'s are taken into account individually in the estimation of e.s.d.'s in distances, angles and torsion angles; correlations between e.s.d.'s in cell parameters are only used when they are defined by crystal symmetry. An approximate (isotropic) treatment of cell e.s.d.'s is used for estimating e.s.d.'s involving l.s. planes.
Refinement. Refinement of *F*^2^ against ALL reflections. The weighted *R*-factor *wR* and goodness of fit *S* are based on *F*^2^, conventional *R*-factors *R* are based on *F*, with *F* set to zero for negative *F*^2^. The threshold expression of *F*^2^ > σ(*F*^2^) is used only for calculating *R*-factors(gt) *etc*. and is not relevant to the choice of reflections for refinement. *R*-factors based on *F*^2^ are statistically about twice as large as those based on *F*, and *R*- factors based on ALL data will be even larger.

### Fractional atomic coordinates and isotropic or equivalent isotropic displacement parameters (Å^2^)

**Table:**

	*x*	*y*	*z*	*U*_iso_*/*U*_eq_	Occ. (<1)
C1	0.31061 (12)	0.03137 (16)	0.35655 (11)	0.0454 (5)	
C2	0.25018 (14)	0.07909 (18)	0.40135 (12)	0.0545 (5)	
H2	0.1976	0.0748	0.3838	0.065*	
C3	0.26725 (16)	0.1332 (2)	0.47205 (13)	0.0651 (6)	
H3	0.2264	0.1653	0.5014	0.078*	
C4	0.34483 (17)	0.1389 (2)	0.49831 (13)	0.0659 (7)	
H4	0.3565	0.1749	0.5456	0.079*	
C5	0.40551 (15)	0.09130 (19)	0.45463 (12)	0.0581 (6)	
H5	0.4578	0.0953	0.4730	0.070*	
C6	0.38971 (13)	0.03773 (17)	0.38393 (11)	0.0475 (5)	
C7	0.45891 (13)	−0.01675 (18)	0.34216 (12)	0.0517 (5)	
C8	0.29231 (12)	−0.04012 (17)	0.28538 (11)	0.0457 (5)	
C9	0.22259 (12)	−0.01536 (16)	0.23476 (11)	0.0455 (5)	
C10	0.19467 (14)	−0.10411 (17)	0.18593 (12)	0.0545 (6)	
H10	0.2202	−0.1759	0.1863	0.065*	
C11	0.13056 (15)	−0.08704 (19)	0.13784 (13)	0.0651 (7)	
H11	0.1124	−0.1474	0.1063	0.078*	
C12	0.09212 (14)	0.02014 (19)	0.13574 (13)	0.0579 (6)	
C13	0.11965 (13)	0.10975 (17)	0.18194 (12)	0.0534 (5)	
H13	0.0949	0.1821	0.1803	0.064*	
C14	0.18427 (13)	0.09142 (17)	0.23073 (12)	0.0506 (5)	
H14	0.2026	0.1523	0.2617	0.061*	
C15	−0.01159 (18)	0.1353 (2)	0.07966 (18)	0.0910 (9)	
H15A	−0.0321	0.1567	0.1298	0.137*	
H15B	−0.0550	0.1278	0.0431	0.137*	
H15C	0.0246	0.1942	0.0619	0.137*	
C16	0.53327 (12)	0.09642 (17)	0.11768 (12)	0.0493 (5)	
C17	0.56149 (13)	−0.00727 (18)	0.14841 (13)	0.0574 (6)	
H17	0.5968	−0.0532	0.1203	0.069*	
C18	0.53659 (13)	−0.04138 (18)	0.22118 (13)	0.0570 (6)	
H18	0.5561	−0.1103	0.2424	0.068*	
C19	0.48288 (12)	0.02494 (17)	0.26360 (11)	0.0469 (5)	
C20	0.45431 (12)	0.12945 (16)	0.23247 (11)	0.0468 (5)	
H20	0.4183	0.1745	0.2603	0.056*	
C21	0.47998 (12)	0.16521 (16)	0.16004 (11)	0.0458 (5)	
C22	0.45933 (13)	0.27577 (17)	0.11513 (11)	0.0513 (5)	
C23	0.50415 (14)	0.25532 (17)	0.03939 (12)	0.0522 (5)	
C24	0.54841 (13)	0.15263 (17)	0.04263 (12)	0.0510 (5)	
C25	0.59410 (15)	0.1189 (2)	−0.02029 (14)	0.0624 (6)	
H25	0.6245	0.0513	−0.0179	0.075*	
C26	0.59413 (15)	0.1866 (2)	−0.08662 (14)	0.0676 (7)	
H26	0.6244	0.1642	−0.1293	0.081*	
C27	0.54984 (16)	0.2870 (2)	−0.09027 (13)	0.0668 (7)	
H27	0.5500	0.3315	−0.1356	0.080*	
C28	0.50491 (15)	0.32279 (19)	−0.02722 (12)	0.0616 (6)	
H28	0.4756	0.3914	−0.0297	0.074*	
C29	0.36895 (14)	0.29072 (19)	0.10095 (13)	0.0606 (6)	
H29A	0.3435	0.3006	0.1512	0.073*	
H29B	0.3607	0.3620	0.0718	0.073*	
C30	0.32668 (15)	0.1934 (2)	0.05806 (15)	0.0724 (7)	
H30A	0.3264	0.1258	0.0919	0.087*	
H30B	0.3582	0.1735	0.0128	0.087*	
C35	0.49149 (16)	0.38429 (18)	0.15852 (13)	0.0639 (7)	
H35A	0.4751	0.4528	0.1295	0.077*	
H35B	0.4662	0.3880	0.2091	0.077*	
C36	0.58109 (18)	0.3899 (2)	0.17088 (16)	0.0799 (8)	
H36A	0.5981	0.3204	0.1985	0.096*	
H36B	0.6068	0.3897	0.1204	0.096*	
O1	0.49806 (10)	−0.09043 (15)	0.37646 (9)	0.0735 (5)	
O2	0.33543 (9)	−0.12374 (12)	0.27163 (9)	0.0585 (4)	
O3	0.02933 (12)	0.02748 (15)	0.08580 (11)	0.0861 (6)	
C31	0.2401 (2)	0.2153 (4)	0.0301 (3)	0.0766 (11)	0.761 (3)
H31A	0.2155	0.1414	0.0169	0.092*	0.761 (3)
H31B	0.2100	0.2493	0.0725	0.092*	0.761 (3)
C32	0.2353 (2)	0.2937 (4)	−0.0388 (2)	0.0987 (13)	0.761 (3)
H32A	0.2638	0.2584	−0.0818	0.118*	0.761 (3)
H32B	0.2616	0.3666	−0.0262	0.118*	0.761 (3)
C33	0.1489 (3)	0.3193 (5)	−0.0653 (3)	0.1081 (15)	0.761 (3)
H33A	0.1180	0.3380	−0.0194	0.130*	0.761 (3)
H33B	0.1495	0.3880	−0.0981	0.130*	0.761 (3)
C34	0.1062 (3)	0.2237 (5)	−0.1092 (3)	0.1270 (18)	0.761 (3)
H34A	0.1351	0.2054	−0.1557	0.190*	0.761 (3)
H34B	0.0533	0.2491	−0.1229	0.190*	0.761 (3)
H34C	0.1029	0.1557	−0.0768	0.190*	0.761 (3)
C31'	0.2566 (7)	0.2666 (10)	0.0262 (10)	0.0766 (11)	0.238 (3)
H31C	0.2759	0.3337	−0.0022	0.092*	0.239 (3)
H31D	0.2227	0.2929	0.0682	0.092*	0.239 (3)
C32'	0.2105 (7)	0.1834 (12)	−0.0288 (8)	0.0987 (13)	0.239 (3)
H32C	0.2423	0.1717	−0.0751	0.118*	0.239 (3)
H32D	0.2060	0.1086	−0.0029	0.118*	0.239 (3)
C33'	0.1279 (8)	0.2210 (15)	−0.0543 (9)	0.1081 (15)	0.239 (3)
H33C	0.0999	0.2540	−0.0103	0.130*	0.239 (3)
H33D	0.0980	0.1538	−0.0724	0.130*	0.239 (3)
C34'	0.1324 (12)	0.3105 (15)	−0.1194 (10)	0.1270 (18)	0.239 (3)
H34D	0.1394	0.3866	−0.0973	0.190*	0.239 (3)
H34E	0.0840	0.3086	−0.1497	0.190*	0.239 (3)
H34F	0.1768	0.2929	−0.1524	0.190*	0.239 (3)
C37	0.6092 (2)	0.4955 (3)	0.21618 (19)	0.0996 (10)	
H37A	0.5891	0.5647	0.1903	0.119*	
H37B	0.5860	0.4930	0.2678	0.119*	
C38	0.6984 (3)	0.5057 (3)	0.2246 (3)	0.1409 (15)	
H38A	0.7212	0.4953	0.1732	0.169*	
H38B	0.7166	0.4410	0.2564	0.169*	
C39	0.7350 (7)	0.6192 (7)	0.2601 (5)	0.163 (3)	0.660 (6)
H39A	0.7902	0.6262	0.2443	0.195*	0.660 (6)
H39B	0.7061	0.6862	0.2401	0.195*	0.660 (6)
C40	0.7307 (5)	0.6179 (9)	0.3470 (5)	0.215 (5)	0.660 (6)
H40A	0.6762	0.6261	0.3628	0.322*	0.660 (6)
H40B	0.7616	0.6812	0.3678	0.322*	0.660 (6)
H40C	0.7517	0.5457	0.3664	0.322*	0.660 (6)
C39'	0.7240 (15)	0.5801 (13)	0.2974 (8)	0.163 (3)	0.340 (6)
H39C	0.7030	0.5476	0.3452	0.195*	0.340 (6)
H39D	0.7816	0.5860	0.3017	0.195*	0.340 (6)
C40'	0.6867 (10)	0.6970 (17)	0.2793 (10)	0.215 (5)	0.340 (6)
H40D	0.6878	0.7104	0.2240	0.322*	0.340 (6)
H40E	0.7162	0.7572	0.3055	0.322*	0.340 (6)
H40F	0.6324	0.6977	0.2968	0.322*	0.340 (6)

### Atomic displacement parameters (Å^2^)

**Table:**

	*U*^11^	*U*^22^	*U*^33^	*U*^12^	*U*^13^	*U*^23^
C1	0.0565 (13)	0.0385 (10)	0.0413 (10)	−0.0018 (9)	0.0009 (9)	0.0056 (9)
C2	0.0606 (14)	0.0524 (12)	0.0503 (12)	−0.0024 (11)	−0.0004 (11)	0.0004 (10)
C3	0.0820 (18)	0.0612 (14)	0.0521 (13)	0.0023 (13)	0.0086 (12)	−0.0026 (11)
C4	0.0913 (19)	0.0623 (14)	0.0440 (12)	−0.0041 (13)	−0.0054 (13)	−0.0053 (11)
C5	0.0689 (15)	0.0567 (13)	0.0485 (12)	−0.0056 (12)	−0.0122 (11)	0.0074 (11)
C6	0.0596 (13)	0.0412 (10)	0.0418 (11)	−0.0014 (10)	−0.0039 (10)	0.0105 (9)
C7	0.0567 (13)	0.0457 (11)	0.0525 (12)	−0.0010 (10)	−0.0096 (10)	0.0095 (10)
C8	0.0511 (12)	0.0395 (10)	0.0465 (11)	−0.0053 (10)	0.0041 (9)	0.0051 (9)
C9	0.0539 (12)	0.0391 (10)	0.0435 (11)	−0.0035 (9)	0.0019 (9)	0.0003 (9)
C10	0.0706 (15)	0.0405 (11)	0.0524 (12)	0.0057 (10)	−0.0056 (11)	−0.0045 (9)
C11	0.0899 (18)	0.0452 (12)	0.0600 (14)	0.0012 (12)	−0.0187 (13)	−0.0115 (10)
C12	0.0688 (15)	0.0513 (12)	0.0533 (13)	0.0004 (11)	−0.0143 (11)	−0.0006 (10)
C13	0.0637 (14)	0.0399 (11)	0.0563 (13)	0.0028 (10)	−0.0036 (11)	−0.0012 (10)
C14	0.0625 (14)	0.0395 (11)	0.0498 (12)	−0.0055 (10)	−0.0019 (10)	−0.0050 (9)
C15	0.097 (2)	0.0810 (19)	0.095 (2)	0.0279 (16)	−0.0355 (17)	−0.0021 (16)
C16	0.0540 (13)	0.0398 (11)	0.0542 (12)	−0.0009 (9)	0.0052 (10)	−0.0011 (9)
C17	0.0576 (14)	0.0476 (12)	0.0671 (14)	0.0115 (10)	0.0102 (11)	0.0009 (11)
C18	0.0578 (13)	0.0455 (12)	0.0678 (15)	0.0107 (10)	−0.0017 (11)	0.0102 (11)
C19	0.0469 (12)	0.0434 (11)	0.0502 (11)	0.0005 (9)	−0.0050 (9)	0.0049 (9)
C20	0.0528 (12)	0.0414 (10)	0.0461 (11)	0.0037 (9)	0.0026 (9)	0.0015 (9)
C21	0.0544 (12)	0.0375 (10)	0.0455 (11)	0.0019 (9)	0.0036 (9)	−0.0005 (9)
C22	0.0715 (15)	0.0397 (11)	0.0428 (11)	0.0078 (10)	0.0101 (10)	0.0029 (9)
C23	0.0669 (14)	0.0436 (11)	0.0461 (11)	−0.0030 (10)	0.0094 (10)	0.0001 (9)
C24	0.0578 (13)	0.0437 (11)	0.0516 (12)	−0.0041 (10)	0.0090 (10)	−0.0040 (9)
C25	0.0687 (15)	0.0533 (13)	0.0654 (15)	−0.0006 (11)	0.0188 (12)	−0.0084 (11)
C26	0.0755 (17)	0.0718 (16)	0.0558 (14)	−0.0148 (14)	0.0234 (12)	−0.0107 (12)
C27	0.0872 (18)	0.0630 (15)	0.0504 (13)	−0.0129 (14)	0.0128 (13)	0.0026 (11)
C28	0.0838 (17)	0.0502 (12)	0.0509 (13)	0.0008 (12)	0.0099 (12)	0.0055 (10)
C29	0.0754 (16)	0.0534 (13)	0.0530 (13)	0.0211 (12)	0.0107 (11)	0.0072 (10)
C30	0.0764 (18)	0.0741 (16)	0.0666 (15)	0.0136 (14)	0.0039 (13)	−0.0011 (13)
C35	0.097 (2)	0.0412 (12)	0.0541 (13)	0.0020 (12)	0.0142 (13)	−0.0002 (10)
C36	0.105 (2)	0.0618 (15)	0.0732 (17)	−0.0115 (15)	0.0058 (16)	−0.0099 (13)
O1	0.0780 (11)	0.0756 (11)	0.0669 (10)	0.0208 (9)	−0.0090 (9)	0.0236 (9)
O2	0.0630 (10)	0.0490 (9)	0.0634 (9)	0.0061 (8)	−0.0038 (8)	−0.0058 (7)
O3	0.1017 (14)	0.0675 (11)	0.0883 (13)	0.0143 (10)	−0.0470 (11)	−0.0115 (9)
C31	0.066 (2)	0.088 (3)	0.076 (2)	−0.007 (2)	0.0060 (19)	−0.003 (3)
C32	0.092 (3)	0.124 (3)	0.080 (2)	0.000 (3)	−0.016 (2)	0.007 (2)
C33	0.101 (3)	0.134 (4)	0.089 (3)	0.019 (3)	−0.024 (2)	−0.017 (3)
C34	0.137 (5)	0.133 (5)	0.110 (3)	−0.003 (4)	−0.024 (3)	−0.013 (4)
C31'	0.066 (2)	0.088 (3)	0.076 (2)	−0.007 (2)	0.0060 (19)	−0.003 (3)
C32'	0.092 (3)	0.124 (3)	0.080 (2)	0.000 (3)	−0.016 (2)	0.007 (2)
C33'	0.101 (3)	0.134 (4)	0.089 (3)	0.019 (3)	−0.024 (2)	−0.017 (3)
C34'	0.137 (5)	0.133 (5)	0.110 (3)	−0.003 (4)	−0.024 (3)	−0.013 (4)
C37	0.125 (3)	0.0766 (19)	0.097 (2)	−0.0186 (19)	−0.008 (2)	−0.0147 (17)
C38	0.157 (4)	0.119 (3)	0.146 (4)	−0.047 (3)	−0.030 (3)	−0.016 (3)
C39	0.180 (7)	0.146 (7)	0.161 (8)	−0.059 (6)	−0.023 (7)	−0.003 (6)
C40	0.137 (6)	0.304 (12)	0.204 (9)	0.032 (6)	−0.027 (5)	−0.155 (9)
C39'	0.180 (7)	0.146 (7)	0.161 (8)	−0.059 (6)	−0.023 (7)	−0.003 (6)
C40'	0.137 (6)	0.304 (12)	0.204 (9)	0.032 (6)	−0.027 (5)	−0.155 (9)

### Geometric parameters (Å, º)

**Table:**

C1—C2	1.389 (3)	C29—H29A	0.9700
C1—C6	1.405 (3)	C29—H29B	0.9700
C1—C8	1.501 (3)	C30—C31'	1.542 (9)
C2—C3	1.390 (3)	C30—C31	1.546 (4)
C2—H2	0.9300	C30—H30A	0.9700
C3—C4	1.374 (3)	C30—H30B	0.9700
C3—H3	0.9300	C35—C36	1.516 (4)
C4—C5	1.381 (3)	C35—H35A	0.9700
C4—H4	0.9300	C35—H35B	0.9700
C5—C6	1.383 (3)	C36—C37	1.515 (3)
C5—H5	0.9300	C36—H36A	0.9700
C6—C7	1.505 (3)	C36—H36B	0.9700
C7—O1	1.220 (2)	C31—C32	1.489 (5)
C7—C19	1.488 (3)	C31—H31A	0.9700
C8—O2	1.227 (2)	C31—H31B	0.9700
C8—C9	1.478 (3)	C32—C33	1.542 (5)
C9—C14	1.387 (3)	C32—H32A	0.9700
C9—C10	1.398 (3)	C32—H32B	0.9700
C10—C11	1.363 (3)	C33—C34	1.510 (5)
C10—H10	0.9300	C33—H33A	0.9700
C11—C12	1.391 (3)	C33—H33B	0.9700
C11—H11	0.9300	C34—H34A	0.9600
C12—O3	1.354 (3)	C34—H34B	0.9600
C12—C13	1.377 (3)	C34—H34C	0.9600
C13—C14	1.379 (3)	C31'—C32'	1.545 (9)
C13—H13	0.9300	C31'—H31C	0.9700
C14—H14	0.9300	C31'—H31D	0.9700
C15—O3	1.420 (3)	C32'—C33'	1.513 (9)
C15—H15A	0.9600	C32'—H32C	0.9700
C15—H15B	0.9600	C32'—H32D	0.9700
C15—H15C	0.9600	C33'—C34'	1.521 (10)
C16—C17	1.385 (3)	C33'—H33C	0.9700
C16—C21	1.400 (3)	C33'—H33D	0.9700
C16—C24	1.464 (3)	C34'—H34D	0.9600
C17—C18	1.376 (3)	C34'—H34E	0.9600
C17—H17	0.9300	C34'—H34F	0.9600
C18—C19	1.390 (3)	C37—C38	1.507 (4)
C18—H18	0.9300	C37—H37A	0.9700
C19—C20	1.398 (3)	C37—H37B	0.9700
C20—C21	1.380 (3)	C38—C39	1.563 (7)
C20—H20	0.9300	C38—C39'	1.570 (9)
C21—C22	1.525 (3)	C38—H38A	0.9700
C22—C23	1.524 (3)	C38—H38B	0.9700
C22—C29	1.542 (3)	C39—C40	1.493 (8)
C22—C35	1.548 (3)	C39—H39A	0.9700
C23—C28	1.381 (3)	C39—H39B	0.9700
C23—C24	1.395 (3)	C40—H40A	0.9600
C24—C25	1.383 (3)	C40—H40B	0.9600
C25—C26	1.378 (3)	C40—H40C	0.9600
C25—H25	0.9300	C39'—C40'	1.513 (10)
C26—C27	1.374 (3)	C39'—H39C	0.9700
C26—H26	0.9300	C39'—H39D	0.9700
C27—C28	1.385 (3)	C40'—H40D	0.9600
C27—H27	0.9300	C40'—H40E	0.9600
C28—H28	0.9300	C40'—H40F	0.9600
C29—C30	1.512 (3)		
			
C2—C1—C6	119.00 (19)	C29—C30—C31	117.7 (2)
C2—C1—C8	121.39 (19)	C29—C30—H30A	107.9
C6—C1—C8	119.11 (18)	C31'—C30—H30A	129.9
C1—C2—C3	120.9 (2)	C31—C30—H30A	107.9
C1—C2—H2	119.6	C29—C30—H30B	107.9
C3—C2—H2	119.6	C31'—C30—H30B	105.3
C4—C3—C2	119.7 (2)	C31—C30—H30B	107.9
C4—C3—H3	120.2	H30A—C30—H30B	107.2
C2—C3—H3	120.2	C36—C35—C22	116.23 (19)
C3—C4—C5	120.1 (2)	C36—C35—H35A	108.2
C3—C4—H4	119.9	C22—C35—H35A	108.2
C5—C4—H4	119.9	C36—C35—H35B	108.2
C4—C5—C6	121.0 (2)	C22—C35—H35B	108.2
C4—C5—H5	119.5	H35A—C35—H35B	107.4
C6—C5—H5	119.5	C37—C36—C35	114.1 (2)
C5—C6—C1	119.3 (2)	C37—C36—H36A	108.7
C5—C6—C7	117.36 (19)	C35—C36—H36A	108.7
C1—C6—C7	123.24 (18)	C37—C36—H36B	108.7
O1—C7—C19	120.8 (2)	C35—C36—H36B	108.7
O1—C7—C6	118.25 (19)	H36A—C36—H36B	107.6
C19—C7—C6	120.76 (17)	C12—O3—C15	118.28 (19)
O2—C8—C9	120.15 (18)	C32—C31—C30	112.9 (3)
O2—C8—C1	117.95 (18)	C32—C31—H31A	109.0
C9—C8—C1	121.84 (18)	C30—C31—H31A	109.0
C14—C9—C10	117.62 (19)	C32—C31—H31B	109.0
C14—C9—C8	124.29 (18)	C30—C31—H31B	109.0
C10—C9—C8	118.06 (18)	H31A—C31—H31B	107.8
C11—C10—C9	121.03 (19)	C31—C32—C33	113.2 (4)
C11—C10—H10	119.5	C31—C32—H32A	108.9
C9—C10—H10	119.5	C33—C32—H32A	108.9
C10—C11—C12	120.4 (2)	C31—C32—H32B	108.9
C10—C11—H11	119.8	C33—C32—H32B	108.9
C12—C11—H11	119.8	H32A—C32—H32B	107.8
O3—C12—C13	124.9 (2)	C34—C33—C32	116.6 (4)
O3—C12—C11	115.41 (19)	C34—C33—H33A	108.1
C13—C12—C11	119.7 (2)	C32—C33—H33A	108.1
C12—C13—C14	119.50 (19)	C34—C33—H33B	108.1
C12—C13—H13	120.2	C32—C33—H33B	108.1
C14—C13—H13	120.2	H33A—C33—H33B	107.3
C13—C14—C9	121.77 (18)	C30—C31'—C32'	104.7 (8)
C13—C14—H14	119.1	C30—C31'—H31C	110.8
C9—C14—H14	119.1	C32'—C31'—H31C	110.8
O3—C15—H15A	109.5	C30—C31'—H31D	110.8
O3—C15—H15B	109.5	C32'—C31'—H31D	110.8
H15A—C15—H15B	109.5	H31C—C31'—H31D	108.9
O3—C15—H15C	109.5	C33'—C32'—C31'	116.8 (12)
H15A—C15—H15C	109.5	C33'—C32'—H32C	108.1
H15B—C15—H15C	109.5	C31'—C32'—H32C	108.1
C17—C16—C21	120.41 (19)	C33'—C32'—H32D	108.1
C17—C16—C24	130.80 (19)	C31'—C32'—H32D	108.1
C21—C16—C24	108.79 (17)	H32C—C32'—H32D	107.3
C18—C17—C16	119.0 (2)	C32'—C33'—C34'	110.8 (13)
C18—C17—H17	120.5	C32'—C33'—H33C	109.5
C16—C17—H17	120.5	C34'—C33'—H33C	109.5
C17—C18—C19	121.37 (19)	C32'—C33'—H33D	109.5
C17—C18—H18	119.3	C34'—C33'—H33D	109.5
C19—C18—H18	119.3	H33C—C33'—H33D	108.1
C18—C19—C20	119.55 (19)	C33'—C34'—H34D	109.5
C18—C19—C7	118.53 (18)	C33'—C34'—H34E	109.5
C20—C19—C7	121.90 (18)	H34D—C34'—H34E	109.5
C21—C20—C19	119.43 (18)	C33'—C34'—H34F	109.5
C21—C20—H20	120.3	H34D—C34'—H34F	109.5
C19—C20—H20	120.3	H34E—C34'—H34F	109.5
C20—C21—C16	120.20 (18)	C38—C37—C36	114.6 (3)
C20—C21—C22	129.18 (18)	C38—C37—H37A	108.6
C16—C21—C22	110.62 (17)	C36—C37—H37A	108.6
C23—C22—C21	100.97 (16)	C38—C37—H37B	108.6
C23—C22—C29	111.91 (17)	C36—C37—H37B	108.6
C21—C22—C29	113.04 (17)	H37A—C37—H37B	107.6
C23—C22—C35	111.23 (18)	C37—C38—C39	119.2 (5)
C21—C22—C35	110.66 (17)	C37—C38—C39'	112.5 (10)
C29—C22—C35	108.90 (17)	C37—C38—H38A	107.5
C28—C23—C24	120.00 (19)	C39—C38—H38A	107.5
C28—C23—C22	128.77 (19)	C39'—C38—H38A	133.0
C24—C23—C22	111.23 (17)	C37—C38—H38B	107.5
C25—C24—C23	120.2 (2)	C39—C38—H38B	107.5
C25—C24—C16	131.5 (2)	C39'—C38—H38B	83.6
C23—C24—C16	108.23 (18)	H38A—C38—H38B	107.0
C26—C25—C24	119.3 (2)	C40—C39—C38	111.1 (6)
C26—C25—H25	120.4	C40—C39—H39A	109.4
C24—C25—H25	120.4	C38—C39—H39A	109.4
C27—C26—C25	120.6 (2)	C40—C39—H39B	109.4
C27—C26—H26	119.7	C38—C39—H39B	109.4
C25—C26—H26	119.7	H39A—C39—H39B	108.0
C26—C27—C28	120.7 (2)	C40'—C39'—C38	102.2 (11)
C26—C27—H27	119.6	C40'—C39'—H39C	111.3
C28—C27—H27	119.6	C38—C39'—H39C	111.3
C23—C28—C27	119.2 (2)	C40'—C39'—H39D	111.3
C23—C28—H28	120.4	C38—C39'—H39D	111.3
C27—C28—H28	120.4	H39C—C39'—H39D	109.2
C30—C29—C22	116.75 (18)	C39'—C40'—H40D	109.5
C30—C29—H29A	108.1	C39'—C40'—H40E	109.5
C22—C29—H29A	108.1	H40D—C40'—H40E	109.5
C30—C29—H29B	108.1	C39'—C40'—H40F	109.5
C22—C29—H29B	108.1	H40D—C40'—H40F	109.5
H29A—C29—H29B	107.3	H40E—C40'—H40F	109.5
C29—C30—C31'	97.0 (5)		
			
C6—C1—C2—C3	0.4 (3)	C20—C21—C22—C29	−57.3 (3)
C8—C1—C2—C3	172.24 (19)	C16—C21—C22—C29	123.32 (19)
C1—C2—C3—C4	−0.5 (3)	C20—C21—C22—C35	65.1 (3)
C2—C3—C4—C5	0.1 (3)	C16—C21—C22—C35	−114.2 (2)
C3—C4—C5—C6	0.3 (3)	C21—C22—C23—C28	175.9 (2)
C4—C5—C6—C1	−0.3 (3)	C29—C22—C23—C28	55.4 (3)
C4—C5—C6—C7	−177.14 (19)	C35—C22—C23—C28	−66.7 (3)
C2—C1—C6—C5	−0.1 (3)	C21—C22—C23—C24	−3.7 (2)
C8—C1—C6—C5	−172.05 (17)	C29—C22—C23—C24	−124.25 (19)
C2—C1—C6—C7	176.58 (18)	C35—C22—C23—C24	113.7 (2)
C8—C1—C6—C7	4.6 (3)	C28—C23—C24—C25	1.0 (3)
C5—C6—C7—O1	56.7 (3)	C22—C23—C24—C25	−179.4 (2)
C1—C6—C7—O1	−120.0 (2)	C28—C23—C24—C16	−177.0 (2)
C5—C6—C7—C19	−118.4 (2)	C22—C23—C24—C16	2.6 (2)
C1—C6—C7—C19	64.9 (3)	C17—C16—C24—C25	1.1 (4)
C2—C1—C8—O2	−143.4 (2)	C21—C16—C24—C25	−177.9 (2)
C6—C1—C8—O2	28.4 (3)	C17—C16—C24—C23	178.8 (2)
C2—C1—C8—C9	33.8 (3)	C21—C16—C24—C23	−0.2 (2)
C6—C1—C8—C9	−154.40 (18)	C23—C24—C25—C26	−1.3 (3)
O2—C8—C9—C14	−160.7 (2)	C16—C24—C25—C26	176.2 (2)
C1—C8—C9—C14	22.2 (3)	C24—C25—C26—C27	0.5 (4)
O2—C8—C9—C10	17.3 (3)	C25—C26—C27—C28	0.6 (4)
C1—C8—C9—C10	−159.81 (19)	C24—C23—C28—C27	0.1 (3)
C14—C9—C10—C11	−1.9 (3)	C22—C23—C28—C27	−179.5 (2)
C8—C9—C10—C11	179.9 (2)	C26—C27—C28—C23	−0.9 (4)
C9—C10—C11—C12	0.8 (4)	C23—C22—C29—C30	55.9 (2)
C10—C11—C12—O3	179.9 (2)	C21—C22—C29—C30	−57.3 (2)
C10—C11—C12—C13	0.7 (4)	C35—C22—C29—C30	179.29 (19)
O3—C12—C13—C14	179.9 (2)	C22—C29—C30—C31'	−155.6 (7)
C11—C12—C13—C14	−1.1 (3)	C22—C29—C30—C31	−169.3 (2)
C12—C13—C14—C9	−0.1 (3)	C23—C22—C35—C36	−50.8 (3)
C10—C9—C14—C13	1.6 (3)	C21—C22—C35—C36	60.6 (3)
C8—C9—C14—C13	179.57 (19)	C29—C22—C35—C36	−174.56 (19)
C21—C16—C17—C18	−0.2 (3)	C22—C35—C36—C37	−177.9 (2)
C24—C16—C17—C18	−179.1 (2)	C13—C12—O3—C15	0.3 (4)
C16—C17—C18—C19	1.1 (3)	C11—C12—O3—C15	−178.8 (2)
C17—C18—C19—C20	−1.0 (3)	C29—C30—C31—C32	74.2 (4)
C17—C18—C19—C7	−179.6 (2)	C31'—C30—C31—C32	39.7 (17)
O1—C7—C19—C18	17.0 (3)	C30—C31—C32—C33	−178.0 (3)
C6—C7—C19—C18	−167.94 (19)	C31—C32—C33—C34	−76.0 (6)
O1—C7—C19—C20	−161.6 (2)	C29—C30—C31'—C32'	173.4 (10)
C6—C7—C19—C20	13.5 (3)	C31—C30—C31'—C32'	−37.0 (12)
C18—C19—C20—C21	−0.1 (3)	C30—C31'—C32'—C33'	166.0 (11)
C7—C19—C20—C21	178.51 (19)	C31'—C32'—C33'—C34'	78.9 (19)
C19—C20—C21—C16	0.9 (3)	C35—C36—C37—C38	−176.5 (3)
C19—C20—C21—C22	−178.3 (2)	C36—C37—C38—C39	171.7 (4)
C17—C16—C21—C20	−0.8 (3)	C36—C37—C38—C39'	−156.0 (7)
C24—C16—C21—C20	178.26 (18)	C37—C38—C39—C40	80.4 (9)
C17—C16—C21—C22	178.58 (19)	C39'—C38—C39—C40	−4 (2)
C24—C16—C21—C22	−2.3 (2)	C37—C38—C39'—C40'	−60.9 (15)
C20—C21—C22—C23	−177.0 (2)	C39—C38—C39'—C40'	49.0 (14)
C16—C21—C22—C23	3.6 (2)		

### Hydrogen-bond geometry (Å, º)

**Table:**

*D*—H···*A*	*D*—H	H···*A*	*D*···*A*	*D*—H···*A*
C26—H26···O2^i^	0.93	2.58	3.470 (3)	160

Symmetry code: (i) −*x*+1, −*y*, −*z*.
